# Transporter-Mediated Hepatic Uptake of EOB-DTPA and BOPTA Is Largely Independent of Chelated Metal

**DOI:** 10.1007/s11307-026-02105-9

**Published:** 2026-05-04

**Authors:** Legend E. Kenney, Christiane L. Mallett, Jeremy M.-L. Hix, Tapas Bhattacharyya, Erik M. Shapiro

**Affiliations:** 1 Department of Radiology, Michigan State University, 846 Service Rd, East Lansing, MI 48824 USA; 2 Department of Physiology, Michigan State University, East Lansing, MI USA; 3 Department of Chemical Engineering and Material Science, Michigan State University, East Lansing, MI USA; 4 Department of Biomedical Engineering, Michigan State University, East Lansing, MI USA; 5 Institute for Quantitative Health Science and Engineering, Michigan State University, East Lansing, MI USA

**Keywords:** Metal chelate, Pharmacology, Imaging

## Abstract

**Purpose:**

Metal chelates play a crucial role in diagnostic imaging and radiotherapy. While gadolinium-based chelates are widely used in MRI, radiometal chelates are increasingly used in nuclear medicine and theranostics. Despite their clinical importance, the extent to which the identity of the coordinated metal influences in vivo chelate pharmacology remains unclear. The goal of this work was to determine whether metal substitution alters transporter-mediated cellular uptake and pharmacological behavior of hepatospecific chelates.

**Procedures:**

Apo-forms of the clinical hepatospecific MRI contrast agents EOB-DTPA and BOPTA were generated and re-chelated with eight different metals (Sc, Y, Pr, Eu, Gd, Tb, Dy, Ho). In vitro transport of these chelates was assessed in cells expressing rodent and human hepatic transporters. In vivo hepatic uptake was evaluated in mice expressing either wild-type or human hepatic transporters. Tissue distribution and clearance were quantified analytically.

**Results:**

In vitro uptake of EOB-DTPA and BOPTA chelates by cells expressing rodent and human transporters showed no statistically significant differences across metals. In vivo studies in mice similarly showed no statistically significant differences in hepatic uptake across metals, with the exception of reduced uptake observed for Sc-EOB-DTPA in wild-type animals. No evidence of free metal accumulation in soft tissue was detected. Chelate clearance via renal and hepatobiliary pathways was similar across metals.

**Conclusions:**

Within the class of trivalent metals examined and for EOB-DTPA and BOPTA chelates, transporter-mediated uptake, biodistribution, and clearance were largely independent of metal identity under the conditions tested. These findings support the use of common chelate scaffolds across multiple metals, while highlighting the importance of ligand structure and transporter interactions in governing pharmacology.

## Introduction

Metal chelates are fundamental to modern medical imaging. In clinical MRI, gadolinium-based chelates remain the gold standard, providing T_1_ contrast enhancement (bright signals) and forming the backbone of diagnostic imaging. Other metals have also been explored, notably manganese. Manganese-based chelates such as Mn-DPDP were used clinically for liver imaging [1], while more recently Mn-PyC3A has demonstrated improved stability and clearance [2]. For research purposes, dysprosium-based chelates have been investigated for MR thermometry in temperature mapping [3], and thulium-based chelates have been applied for pH mapping [4].

Beyond MRI, metal chelates are increasingly important in multimodality imaging. A single chelate scaffold can be used with different metals to enable complementary techniques. For example, gadolinium- and yttrium-based versions of the same chelate can be paired for simultaneous PET/MRI [5]. The Gd complex provides high-resolution anatomical and functional information, while the ⁸⁶Y complex offers quantitative PET signal with direct correlation to tracer concentration.

This versatility positions chelates as adaptable platforms for multimodal imaging and theranostic applications. By interchanging metals within a common ligand, diagnostic and therapeutic agents can be designed as matched pairs, aiming for equal biodistribution with complementary readouts. A prominent example is the use of ⁶⁸Ga- and ^177^Lu-DOTATATE: the ⁶⁸Ga complex enables sensitive PET imaging of somatostatin receptor–positive tumors, while the ^177^Lu complex provides targeted radiotherapy to the same lesions [6]. These matched pairs highlight how metal chelation chemistry can bring diagnostic imaging and therapy together within a single molecular design.

The extent to which metal substitution preserves pharmacology across imaging and therapeutic applications has not been systematically established. Although metal chelates are often treated as interchangeable platforms, substitution of the coordinated metal could plausibly influence pharmacology. Differences in ionic radius, coordination geometry, hydration number, and metal–ligand dynamics may alter chelate conformation, charge distribution, and interactions with transporters or proteins. These considerations suggest that metal identity could, in principle, affect transporter recognition and in vivo behavior. A prior study by Krause and colleagues provided an important foundation by showing that EOB-DTPA maintains consistent pharmacology across several lanthanide complexes in rodents, suggesting that ligand–organ interactions may dominate over metal identity [7]. However, their work was limited to hepatic uptake in rodents, leaving open key questions about transporter-level mechanisms and possible species drift. Species differences are particularly relevant for hepatospecific agents. For example, Gd-BOPTA exhibits notable variation in uptake and clearance between rodents and humans [8], and similar effects have been reported for other experimental metal chelates, such as Cu-EOB-NOTA [9].

In this work, we extend these observations to a second hepatospecific chelate, BOPTA, and to a broader set of metals, including scandium and yttrium, which are relevant for PET/MRI applications. By combining in vitro assays in cells expressing rodent and human OATPs with in vivo studies in mice expressing both mouse and human hepatic transporters, we evaluate transporter-mediated uptake and test for species drift effects. By examining two clinically relevant scaffolds across multiple metals and species, our goal was to assess the generalizability of metal-independence in chelate pharmacology.

## Materials and Methods

### Chelate Synthesis

Chelates were generated from clinical products Gd-EOB-DTPA (Eovist) or Gd-BOPTA (Multihance). Metal sources were: GdCl_3_.6H_2_O, EuCl_3_.6H_2_O, TbCl_3_.6H_2_O, HoCl_3_.6H_2_O, DyCl_3_.6H_2_O, ScCl_3_.6H_2_O, and YCl_3_.6H_2_O, all from Sigma Aldrich. PrCl_3_.5H_2_O was from Strem Chemicals, Newburyport, MA.

**Fig. 1:**
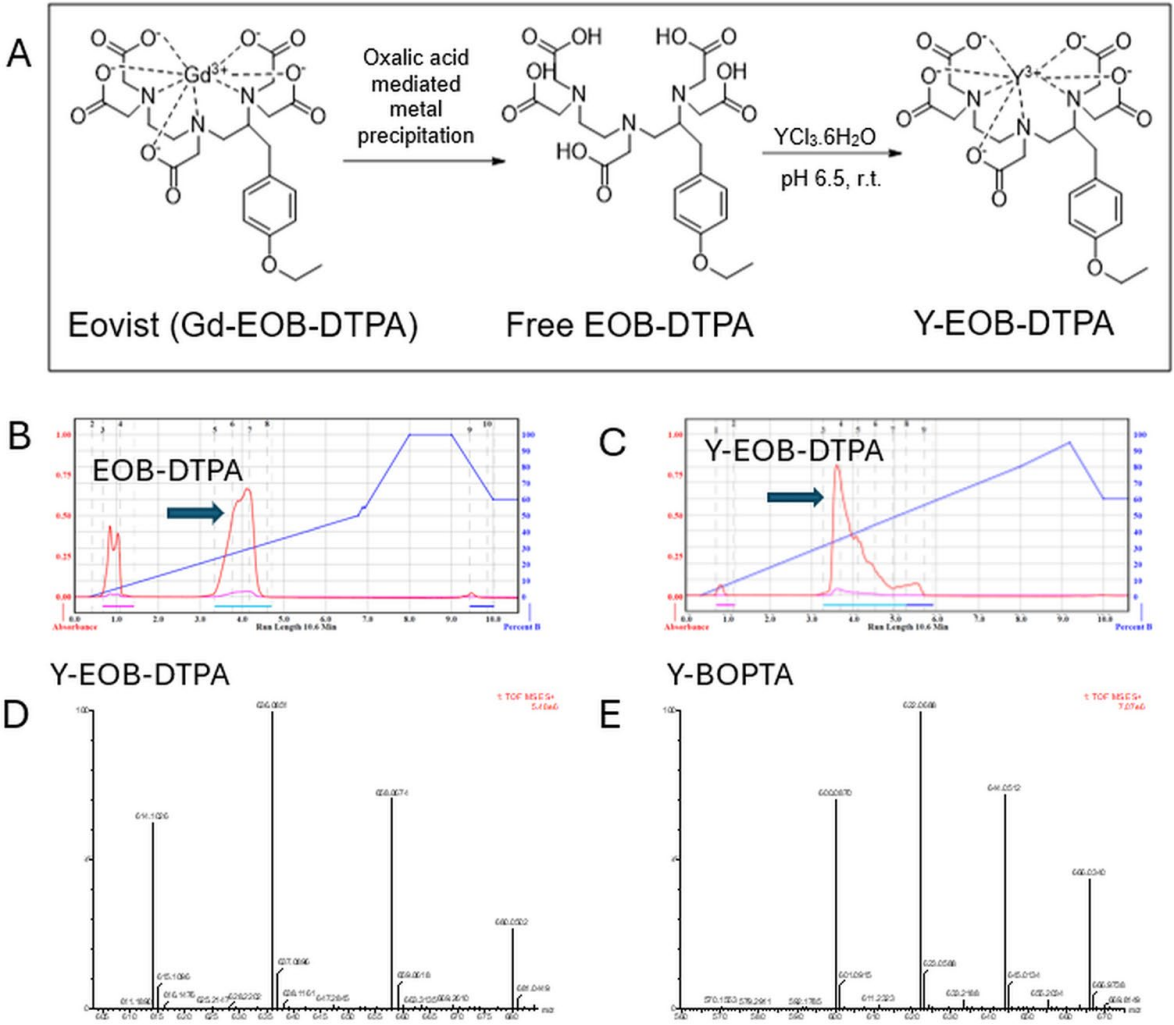
Synthesis of metal chelates. **A**) Chemical schematic of the synthesis of Y-EOB-DTPA from Gd-EOB-DTPA. This generalized procedure was carried out for all chelates, for both EOB-DTPA and BOPTA. **B**, **C**) MPLC traces for the empty EOB-DTPA chelate (**B**) and Y-EOB-DTPA (**C**). **D**, **E**) Mass spec for Y-EOB-DTPA (**D**) and Y-BOPTA (**E**) showing chelated product and numerous sodium adducts.

Following a procedure reported by Patrick, et al., [10] the first step involved the oxalic acid assisted de-complexation of Gd-EOB-DTPA or Gd-BOPTA to separate out the EOB-DTPA or BOPTA ligand and Gd-oxalate (Fig. 1A). In a typical procedure, 52 mg of oxalic acid dihydrate (0.4 mmol) was dissolved in 0.6 mL DI water to obtain a clear solution. Next, 0.4 mL Gd-EOB-DTPA (0.1 mmol) was added to this solution resulting in immediate precipitation of white Gd-oxalate. The reaction mixture was centrifuged and the supernatant that contained the free EOB-DTPA ligand was pipetted out and lyophilized to obtain a crystalline solid. This solid was dissolved in 1 mL water and purified on an MPLC (medium-pressure liquid chromatography) column (C18 reverse phase column, 30 g; solvent system: A = 25 mM ammonium formate; B = methanol; eluent run composition: 0% methanol to 100% methanol, compound was eluted at 25–35% methanol in ~ 4 min, flow rate: 40 mL/min) (Fig. 1B). The product was recovered as a white solid via solvent removal and lyophilization. Mass spectrometry was used to verify the product. The same procedure was used to generate free BOPTA from Gd-BOPTA, though Gd-BOPTA is 2X concentrated versus Gd-EOB-DTPA.

To generate the metal EOB-DTPA and BOPTA complexes, the free ligands were first dissolved in 5 mL DI water, resulting in pH ~ 2–3. Metal chlorides were then added in a 1:1 proportion, further reducing the pH. Using 0.1 M NaOH, the pH of the solution was slowly brought to pH 6–6.5, and the reaction was stirred overnight at room temperature to ensure complete complexation. The products were then recovered via filtration and lyophilization. Final products were purified by MPLC under the same conditions as above and lyophilized to dryness (Fig. 1C). Chelate formation was validated by mass spectrometry and metal content was measured using ICP-QQQ-MS. Quantification was performed using multi-element calibration standards, and measurements were validated across the concentration range used in this study.

### *In Vitro *Transport Studies

Unmodified HEK293 cells and cells stably expressing rat OATP1B2 or human OATP1B3 [11–13] were maintained in DMEM supplemented with 10% fetal bovine serum and 1% penicillin/streptomycin at 37 °C, 5% CO₂. Cocktails containing all eight EOB-DTPA based metal chelates or BOPTA based chelates (each individual chelate at 0.1 mM) was prepared in culture medium. This enabled multiplexed, simultaneous quantification of each chelate in a single sample. Keeping the total chelate concentration at 0.8 mM ensured the concentration was below the reported K_m_ for hepatic transporters [14]. Cells were incubated (*n* = 3 replicates) with each cocktail for 10 min, washed three times with cold PBS, harvested, dried, and digested in 70% HNO₃. Metal content was quantified by ICP-QQQ-MS, with calibration against multi-element ICP standards containing all of the metals under investigation. ICP-QQQ-MS validated that each chelate in the multi-metal cocktail was present at the target concentration of 0.1 mM ± 5%, confirming dosing accuracy for both in vitro and in vivo studies.

### *In Vivo *Pharmacology

All animal procedures were in performed according to an approved MSU IACUC protocol in accordance with AALAS guidelines for animal experiments. Animal experiments were performed in wild-type mice and in mice expressing human OATP1B1 and OATP1B3 on a rodent OATP knockout background (OATP1B1/1B3 mice) [15]. Wild-type mice (*n* = 3 per group) were injected via tail vein with 250 µL of either the 8-metal EOB-DTPA-based cocktail or BOPTA-based cocktail (each individual chelate at 0.1 mM). OATP1B1/1B3 mice (*n* = 3) were injected via tail vein with 250 µL of only the 8-metal EOB-DTPA-based cocktail. After 10 min, the time previously determined as peak liver uptake [16], mice were perfused with saline, and liver, kidney, and quadriceps tissues were harvested. Samples were microwave-digested in 70% HNO₃ for 2 h and analyzed by ICP-QQQ-MS.

Clearance studies were performed using a combined formulation of Gd-EOB-DTPA and Y-EOB-DTPA. Wild-type and human OATP1B1/1B3 knock-in mice (*n* = 3 per group) were injected intravenously with 250 µL of agent (0.1 mM). Mice were housed individually in metabolic cages for 24 h following injection. Urine and feces were collected separately, dried, and digested in 70% nitric acid. Renal and hepatobiliary clearance were quantified by ICP-QQQ-MS as the fraction of recovered metal excreted in urine versus feces.

### Data Analysis

For each experiment, uptake was measured in three independent cell experiments or three animals, with all eight metals assessed in every case. Because each sample was measured across all metals, the data were analyzed using a repeated-measures framework, treating metal as a within-subject factor.

To account for differences in overall uptake between samples, we used within-subject statistical approaches. The primary analysis consisted of repeated-measures ANOVA with metal as the within-subject factor. When assumptions were uncertain or confirmation was needed, we additionally applied the Friedman test as a nonparametric alternative. When a significant overall effect of metal was observed, post-hoc comparisons were performed using paired Wilcoxon signed-rank tests to compare metals within the same sample. Holm correction was applied to adjust for multiple testing, and metal chelates were considered distinct only if they remained significant after correction.

Variability between samples was further explored by visually examining uptake profiles across metals. For visualization purposes only, some plots used within-sample normalization (demeaning); all statistical analyses were conducted on the raw data. Analyses were performed in Python (v3.11) using *pandas, scipy, and statsmodels*, with statistical significance defined as *p* < 0.05.

## Results

### Chelate Chemistry and Validation

Metal chelation of EOB-DTPA and BOPTA with each of the 8 metals was successful, with mass spectrometry confirming weights (Figs. 1D and 1E, for example; also Table 1) and ICP-QQQ-MS measuring metal content. Mass spectrometry was run in ES + mode, and so both Pr and Sc chelates are disodium adducts. All other chelates mass are H^+^.

**Table 1:** Mass spectrometry validation of metal chelates

Chemical	Predicted m/z	Measured m/z (H +)
Gd-EOB-DTPA	682.10	683.18
Pr-EOB-DTPA	665.10	710.01 (2 × Na—H)
Sc-EOB-DTPA	569.14	614.12 (2 × Na—H)
Tb-EOB-DTPA	683.10	684.12
Eu-EOB-DTPA	677.09	678.12
Ho-EOB-DTPA	689.10	690.13
Y-EOB-DTPA	613.08	614.10
Dy-EOB-DTPA	688.10	689.13
Gd-BOPTA	668.10	669.12
Pr-BOPTA	651.08	696.06 (2 × Na—H)
Sc-BOPTA	555.13	600.10 (2 × Na—H)
Tb-BOPTA	669.10	670.11
Y-BOPTA	599.08	600.09
Eu-BOPTA	663.09	664.10
Ho-BOPTA	675.10	676.12
Dy-BOPTA	674.10	675.11

Predicted and measured m/z values for EOB-DTPA and BOPTA complexes with each metal, confirming successful chelation

### *In-vitro* Transport Studies

**Fig. 2:**
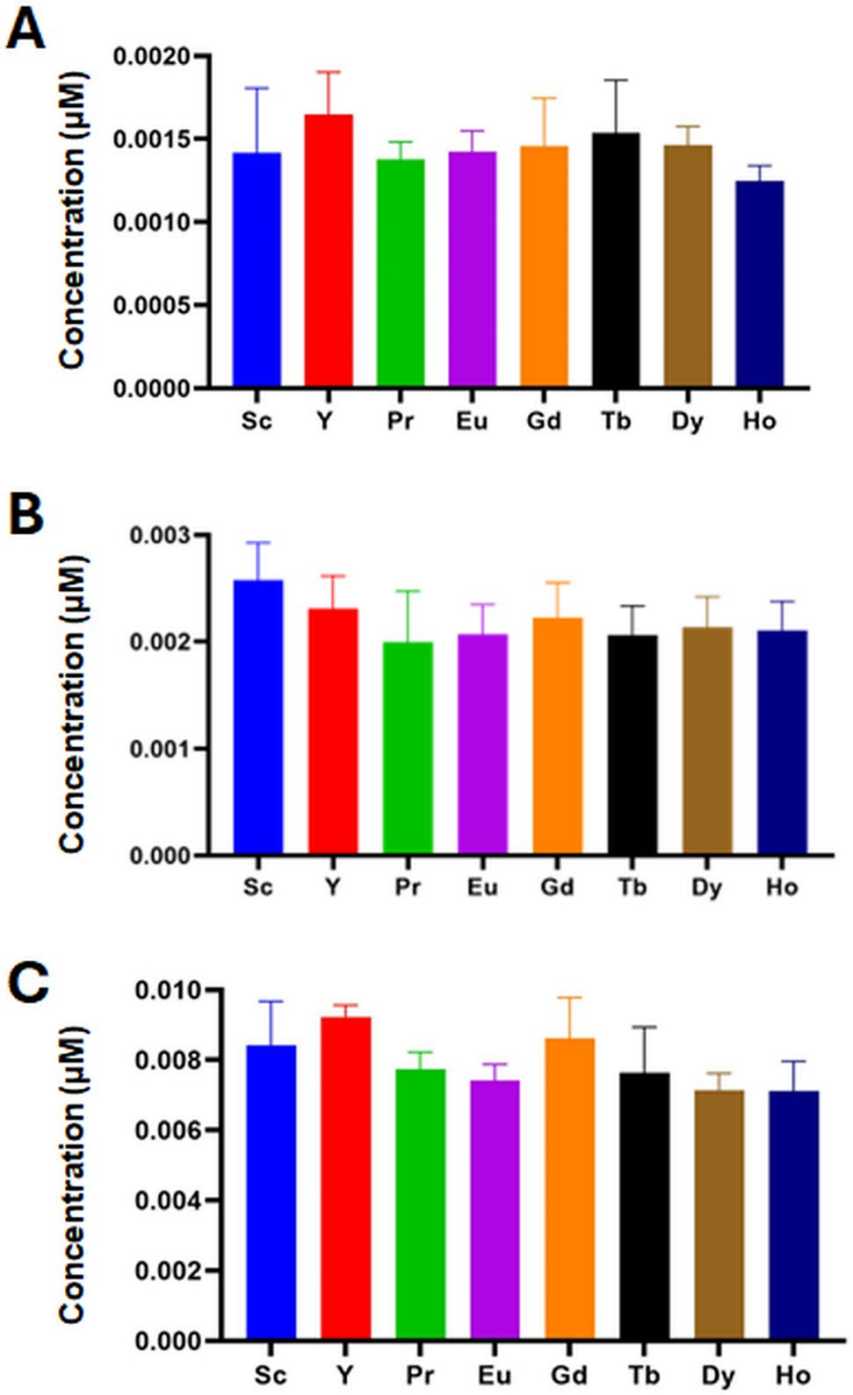
In vitro uptake of metal chelates. (**A**) Uptake of BOPTA-based chelates in HEK cells expressing OATP1B2. (**B**) Uptake of EOB-DTPA-based chelates in HEK cells expressing OATP1B2. (**C**) Uptake of EOB-DTPA-based chelates in HEK cells expressing OATP1B3. Data are shown as mean ± SD (*n* = 3). No statistically significant differences were detected across metals.

In cells expressing rat OATP1B2, there was no statistically significant difference in the relative uptake of any metal chelate, either BOPTA (Fig. 2A) or EOB-DTPA (Fig. 2B). In cells expressing OATP1B3, there was no statistically significant difference in the relative uptake of any metal EOB-DTPA chelate (Fig. 2C).

### *In-vivo* Pharmacology

#### Wild Type Mice

**Fig. 3:**
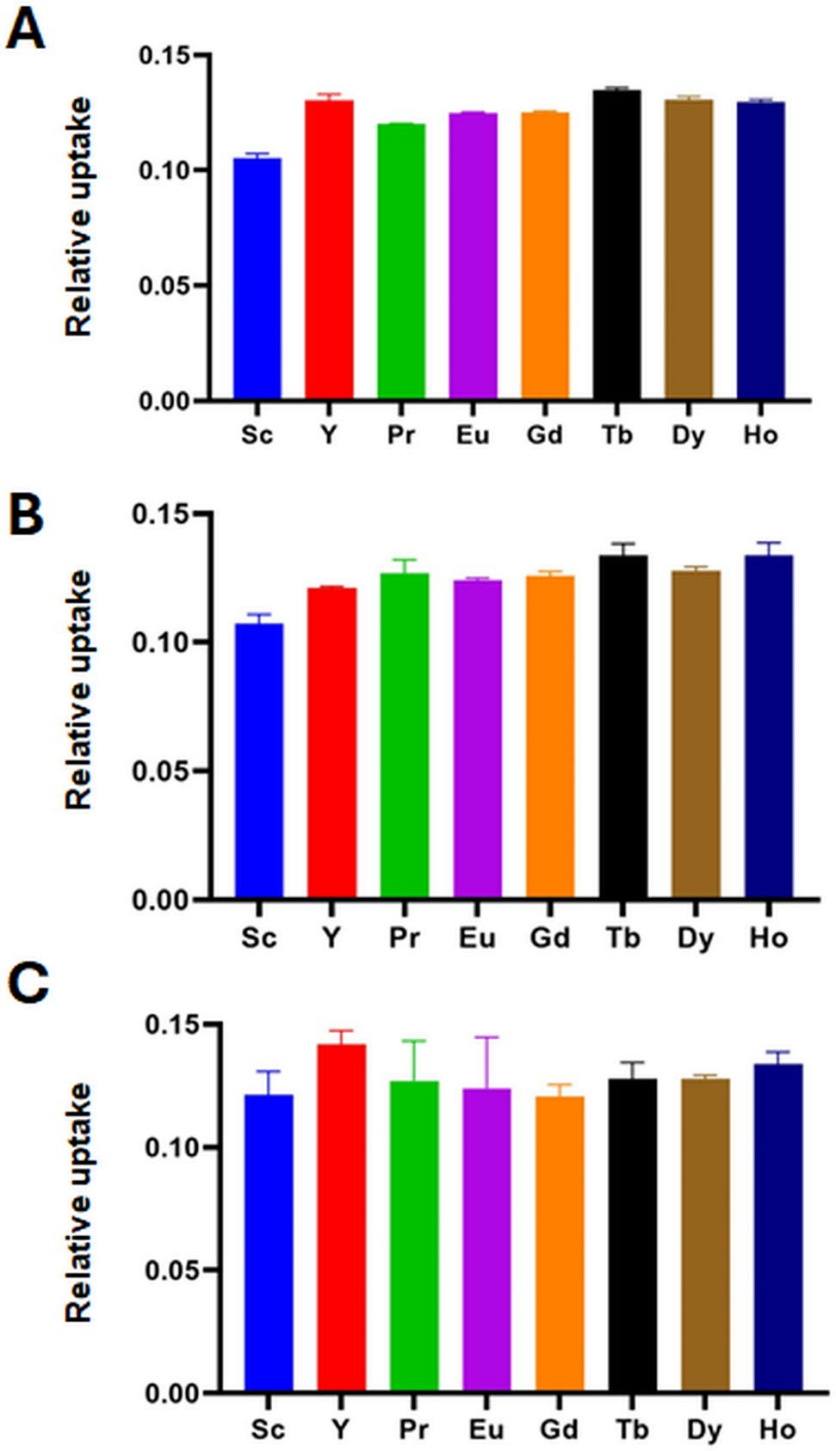
In vivo uptake of metal chelates. (**A**) Relative hepatic uptake (fraction of total accumulated) of BOPTA-based chelates in wild type mice expressing OATP1B2. (**B**) Relative hepatic uptake of EOB-DTPA-based chelates in wild type mice expressing OATP1B2. (**C**) Relative hepatic uptake of EOB-DTPA-based chelates in transgenic mice expressing OATP1B1 and OATP1B3. Data are shown as mean ± SD (*n* = 3). No statistically significant differences were detected across metals, except for reduced uptake of Sc-EOB-DTPA in wild-type mice.

Since the 8 chelates were all injected as a cocktail, for this analysis Fig. 3 shows chelate uptake as a percent total accumulated per animal. For BOPTA, there was no statistically significant difference in the relative uptake of any metal chelate (Fig. 3A). For EOB-DTPA chelates, Sc-EOB-DTPA exhibited significantly reduced uptake relative to other metals (Fig. 3B). Importantly, no significant accumulation of free metals was detected in bone or soft tissue, nor in kidney, indicating that the chelates remained intact and were efficiently cleared.

#### Knock-In Mice

There was no statistically significant difference in the relative uptake of any metal EOB-DTPA chelate. Figure 3C shows the uptake data for each chelate as a percent total chelate per animal.

#### Clearance Study

Both Gd-EOB-DTPA and Y-EOB-DTPA were excreted in a roughly 50/50 split between hepatic (biliary) and renal pathways, in both wild type and human OATP knock-in mice, consistent with known clearance mechanisms for Gd-EOB-DTPA.

## Discussion

This study demonstrates that the transport-mediated hepatic uptake of EOB-DTPA and BOPTA complexes is largely independent of the metal species chelated. Using in vitro and in vivo experimentation, we observed no statistically significant differences in uptake across metals in both rodent and human OATP transporter systems, with only minor deviations observed for scandium. These findings suggest that the pharmacokinetics of these metal chelates are governed more by the chelate structure and transporter interaction than by the metal ion itself. Importantly, these observations primarily reflect OATP-mediated hepatic uptake, and other transport processes, including efflux pathways, may also contribute to overall pharmacokinetics but were not directly assessed in this study. The multiplexed cocktail design enabled direct comparison of multiple chelates within the same biological system. However, although total chelate concentration was maintained below reported K_m_ values, simultaneous exposure to multiple substrates may introduce some degree of competition among substrates for transporter binding. This design preserves relative comparisons across metals but may reduce sensitivity to detect subtle differences in transporter affinity.

Although metal chelates are often treated as interchangeable platforms, there are several chemical and biological reasons why substitution of the coordinated metal could plausibly alter in vivo pharmacology. Differences in ionic radius, coordination geometry, and hydration number across metals can subtly influence chelate conformation, surface polarity, and interaction with solvent or proteins. Even when thermodynamic stability is high, variations in kinetic stability or metal–ligand bond dynamics could affect circulation time or tissue retention. In addition, transporter-mediated uptake depends on molecular shape and charge distribution rather than the metal ion, raising the possibility that metal-dependent perturbations could influence recognition or transport efficiency, particularly across species with different transporter properties [17–19]. The reduced uptake observed for Sc-EOB-DTPA may reflect subtle differences in coordination chemistry relative to the lanthanide series. Scandium has a smaller ionic radius and may exhibit differences in coordination geometry or ligand interaction, which could alter chelate conformation or charge distribution and influence transporter recognition. Although speculative, this observation may represent a boundary condition for metal independence within this class of chelates and highlights that even within trivalent systems, subtle metal-dependent effects may arise.

The metal-independence observed in this study can be interpreted within the context of conserved coordination chemistry. The metals examined here, lanthanides, yttrium, and scandium, share a common trivalent charge and favor high coordination numbers, allowing EOB-DTPA and BOPTA to adopt similar coordination geometries and molecular architectures across substitutions [20]. Under these conditions, different metals might produce only modest perturbations to chelate conformation, hydration, and charge distribution, consistent with the highly similar transporter-mediated uptake, biodistribution, and clearance observed in vivo.

By contrast, substitution with metals that exhibit substantially different coordination preferences would not necessarily be expected to preserve pharmacology. Metals such as gallium, which favor lower coordination numbers and more rigid coordination geometries, could induce reorganization or partial disengagement of DTPA-based ligands, potentially altering chelate shape, electrostatics, and interactions with biological targets [21]. Although we did not examine these effects here, this point marks an important limitation to metal-independence and emphasizes the need to consider coordination chemistry when extending pharmacological findings across different metal substitutions.

This study has several limitations. First, the sample size was limited (*n* = 3), reducing statistical power to detect small differences between metals and limiting the ability to formally demonstrate equivalence. Second, only trivalent metals with similar coordination chemistry were examined, limiting generalization to metals with different coordination preferences. Third, only two chelate scaffolds were evaluated. Fourth, uptake was assessed at a single early time point, and potential differences in later pharmacokinetics cannot be excluded. Finally, the multiplexed cocktail design may reduce sensitivity to detect subtle differences in transporter affinity.

## Conclusion

This study demonstrates that, within the class of trivalent metals examined and for EOB-DTPA and BOPTA scaffolds, transporter-mediated hepatic uptake and overall pharmacological behavior are largely independent of the coordinated metal under the conditions tested. Across multiple metals, two chelate scaffolds, and both rodent and human transporter systems, uptake, biodistribution, and clearance were primarily governed by ligand architecture and transporter interactions. These findings support the use of common chelate scaffolds across metals for imaging applications while highlighting the importance of coordination chemistry when extending these results beyond the systems studied.

## References

[1] King LJ, Burkill GJC, Scurr ED, Vlavianos P, Murray-Lyons I, Healy JC (2002) MnDPDP enhanced magnetic resonance imaging of focal liver lesions. Clin Radiol 57(12):1047–1057 12475527 10.1053/crad.2002.1117

[2] Gale EM, Wey HY, Ramsay I, Yen YF, Sosnovik DE, Caravan P (2018) A manganese-based alternative to gadolinium: contrast-enhanced MR angiography, excretion, pharmacokinetics, and metabolism. Radiology 286(3):865–872 29117483 10.1148/radiol.2017170977 PMC5831267

[3] Shapiro EM, Borthakur A, Shapiro MJ, Reddy R, Leigh JS (2002) Fast MRI of RF heating via phase difference mapping. Magn Reson Med 47(3):492–498 11870836 10.1002/mrm.10067 PMC2855824

[4] Coman D, de Graaf RA, Rothman DL, Hyder F (2013) In vivo three-dimensional molecular imaging with biosensor imaging of redundant deviation in shifts (BIRDS) at high spatiotemporal resolution. NMR Biomed 26(11):1589–95 23881869 10.1002/nbm.2995 PMC3800475

[5] Le Fur M, Rotile NJ, Correcher C, Clavijo Jordan V, Ross AW, Catana C, Caravan P (2020) Yttrium-86 is a positron emitting surrogate of Gadolinium for noninvasive quantification of whole-body distribution of Gadolinium-based contrast agents. Angew Chem Int Ed Engl 59(4):1474–1478 31750991 10.1002/anie.201911858 PMC7027945

[6] Ortega C, Wong RKS, Schaefferkoetter J, Veit-Haibach P, Myrehaug S, Juergens R, Laidley D, Anconina R, Liu A, Metser U (2021) Quantitative (68)Ga-DOTATATE PET/CT parameters for the prediction of therapy response in patients with progressive metastatic neuroendocrine tumors treated with (177)Lu-DOTATATE. J Nucl Med 62(10):1406–1414 33579805 10.2967/jnumed.120.256727 PMC8724892

[7] Krause W, Schuhmann-Giampieri G, Bauer M, Press WR, Muschick P (1996) Ytterbium- and dysprosium-EOB-DTPA. A new prototype of liver-specific contrast agents for computed tomography. Invest Radiol 31(8):502–511 8854197 10.1097/00004424-199608000-00006

[8] Kirchin MA, Pirovano GP, Spinazzi A (1998) Gadobenate dimeglumine (Gd-BOPTA). An overview. Invest Radiol 33(11):798–809 9818314 10.1097/00004424-199811000-00003

[9] Fan J, Ramulu BJ, Mallett CL, Kenney LE, Kauffman N, Bhattacharyya T, Sabbaghan M, Singh S, Zinn KR, Shapiro EM (2025) Species-specific hepatic uptake of [64Cu]Cu-EOB-NOTA, a newly designed hepatospecific PET agent. Mol Imaging Biol. 10.1007/s11307-025-02009-0 40304857 10.1007/s11307-025-02009-0 PMC12162674

[10] Patrick PS, Hammersley J, Loizou L, Kettunen MI, Rodrigues TB, Hu DE, Tee SS, Hesketh R, Lyons SK, Soloviev D, Lewis DY, Aime S, Fulton SM, Brindle KM (2014) Dual-modality gene reporter for in vivo imaging. Proc Natl Acad Sci U S A 111(1):415–420 24347640 10.1073/pnas.1319000111 PMC3890795

[11] Bhattacharyya T, Mallett CL, Hix JM, Shapiro EM (2025) DCE-MRI detects OATP-expressing transplanted cells using clinical doses of Gadolinium contrast agent. Mol Imaging Biol 27(2):184–191 39904956 10.1007/s11307-025-01986-6

[12] Shapiro JA, Bhattacharyya T, Squire LA, Mallett CL, Hix JM, Kenney LE, Aguirre A, Shapiro EM (2025) Go fish! hepatic uptake of clinical hepatospecific Gadolinium-based MRI contrast agents in zebrafish is similar to humans. Mol Imaging Biol. 10.1007/s11307-025-02023-2 40461717 10.1007/s11307-025-02023-2 PMC12405032

[13] Bhattacharyya T, Mallett CL, Shapiro EM (2024) MRI-based cell tracking of OATP-expressing cell transplants by pre-labeling with Gd-EOB-DTPA. Mol Imaging Biol 26(2):233–239 38448775 10.1007/s11307-024-01904-2

[14] Leonhardt M, Keiser M, Oswald S, Kuhn J, Jia J, Grube M, Kroemer HK, Siegmund W, Weitschies W (2010) Hepatic uptake of the magnetic resonance imaging contrast agent Gd-EOB-DTPA: role of human organic anion transporters. Drug Metab Dispos 38(7):1024–1028 20406852 10.1124/dmd.110.032862

[15] Mir FF, Tomaszewski RP, Shuboni-Mulligan DD, Mallett CL, Hix JML, Ether ND, Shapiro EM (2019) Chimeric mouse model for MRI contrast agent evaluation. Magn Reson Med 82(1):387–394 30874333 10.1002/mrm.27730 PMC6491262

[16] Shuboni-Mulligan DD, Parys M, Blanco-Fernandez B, Mallett CL, Schnegelberger R, Takada M, Chakravarty S, Hagenbuch B, Shapiro EM (2019) Dynamic contrast-enhanced MRI of OATP dysfunction in Diabetes. Diabetes 68(2):271–280 30487262 10.2337/db18-0525 PMC6341305

[17] Kunze A, Huwyler J, Camenisch G, Poller B (2014) Prediction of organic anion-transporting polypeptide 1B1- and 1B3-mediated hepatic uptake of statins based on transporter protein expression and activity data. Drug Metab Dispos 42(9):1514–1521 24989890 10.1124/dmd.114.058412

[18] Karlgren M, Vildhede A, Norinder U, Wisniewski JR, Kimoto E, Lai Y, Haglund U, Artursson P (2012) Classification of inhibitors of hepatic organic anion transporting polypeptides (OATPs): influence of protein expression on drug-drug interactions. J Med Chem 55(10):4740–4763 22541068 10.1021/jm300212s PMC3361267

[19] Mardikoraem M, Eaves JN, Belecciu T, Pascual N, Aljets A, Hagenbuch B, Shapiro EM, Orlando BJ, Woldring DR (2025) Predicting inhibitors of OATP1B1 via heterogeneous OATP-ligand interaction graph neural network (HOLIgraph). J Cheminform 17(1):69 40325434 10.1186/s13321-025-01020-5 PMC12054207

[20] Schmitt-Willich H, Brehm M, Ewers CL, Michl G, Muller-Fahrnow A, Petrov O, Platzek J, Raduchel B, Sulzle D (1999) Synthesis and physicochemical characterization of a new gadolinium chelate: the liver-specific magnetic resonance imaging contrast agent Gd-EOB-DTPA. Inorg Chem 38(6):1134–1144 11670895 10.1021/ic981072i

[21] Greiser J, Niksch T, Weigand W, Freesmeyer M (2016) Investigations on the Ga(III) complex of EOB-DTPA and its ^68^Ga radiolabeled analogue. J Vis Exp. 10.3791/54334 27584545 10.3791/54334 PMC5091902

